# Acetyl-DL-leucine improves gait variability in patients with cerebellar ataxia—a case series

**DOI:** 10.1186/s40673-016-0046-2

**Published:** 2016-04-12

**Authors:** Roman Schniepp, Michael Strupp, Max Wuehr, Klaus Jahn, Marianne Dieterich, Thomas Brandt, Katharina Feil

**Affiliations:** Department of Neurology, University of Munich, Campus Großhadern, Marchioninistrasse 15, Munich, 81377 Germany; German Center for Vertigo and Balance Disorders (DSGZ), University of Munich, Campus Großhadern, Marchioninistrasse 15, Munich, 81377 Germany; Schoen Klinik Bad Aibling, Neurologie, Kolbermoorer Strasse 72, Bad Aibling, 83043 Germany; Institute of Clinical Neurosciences, University of Munich, Campus Großhadern, Marchioninistrasse 15, Munich, 81377 Germany

**Keywords:** Gait variability, Acetyl-DL-leucine, Cerebellar ataxia, Speed dependency, symptomatic treatment

## Abstract

Acetyl-DL-leucine is a modified amino acid that was observed to improve ataxic symptoms in patients with sporadic and hereditary forms of ataxia. Here, we investigated the effect of the treatment with Acetyl-DL-leucine on the walking stability of patients with cerebellar ataxia (10x SAOA, 2x MSA-C, 2x ADA, 1x CACNA-1A mutation, 2x SCA 2, 1x SCA 1). Treatment with Acetyl-DL-leucine (500 mg; 3-3-4) significantly improved the coefficient of variation of stride time in 14 out of 18 patients. Moreover, subjective ambulatory scores (FES-I and ABC) and the SARA scores were also improved under treatment. Further prospective studies are necessary to support these class III observational findings.

## Introduction

Mobility impairments in patients with cerebellar ataxia (CA) are common. They are associated with a higher risk of falls, which often result in an increased fear of falling as well as in serious injuries [1]. Thus, symptomatic treatment options for patients with CA should aim to reduce the occurrence of falls by improving their dynamic balance. Gait variability measures which are linked to dynamic walking stability were found to be associated to falls in CA [2], thereby providing a novel and also quantitative measure for the assessment of ataxic gait disorders.

Acetyl-DL-leucine, a modified amino acid, was observed to improve ataxic symptoms in patients with sporadic and hereditary forms of ataxia [3], as well as in patients with Niemann-Pick Type C [4]. This effect was not found in a similar study performed in patients with sporadic, degenerative ataxia only [5].

Here we report on the effect of acetyl-DL-leucine on temporal gait variability in 18 patients with cerebellar ataxia (12 sporadic, 6 hereditary ataxia).

## Methods

Patients were treated with acetyl-DL-leucine 5 g/d without titration (500 mg tablets of Tanganil™) for at least 4 weeks. Indication for the treatment with acetyl-DL-leucine were the presence of cerebellar symptoms and abnormal findings in the domain “gait” of the Scale and Assessment of Ataxia (SARA). Exclusion criteria were concomitant diseases that affect locomotion. Twelve out of the 18 enrolled subjects received physiotherapeutic treatments with a weekly intensity of 20–60 min already before the pharmacological off-label treatment and also during it. Physiotherapy was not started new during the off-label treatment with acetyl-DL-leucine. All patients gave their informed consent for the compassionate off-label use of acetyl-DL-leucine. The series was conducted by individual treatment efforts of patients after they had given informed consent. Eight of these patients had been part of our previous case series [3]. Treatment of patients took place from 2013 to 2014 at the Department of Neurology and at the German Center for Vertigo and Balance Disorders. We performed gait analysis using a pressure-sensitive carpet (GAITRite®, CIR System, Havertown, USA; sensor area 6.70 m, sampling rate 120 Hz). The walking pattern of all patients was recorded at three different walking speeds (slow, preferred, and maximal fast). Each speed was tested four times. Gait velocity and temporal gait variability quantified by the coefficient of variation (CV) of stride time were analyzed. The tests were performed before starting of treatment and during treatment at time point 1 (7–14 days) and time point 2 (28–42 day). A repeated measure ANOVA (rmANOVA) with the covariate “walking speed” and the inner-subject factor “time point” was performed. A Sheffé posthoc analysis was performed.

## Results

Clinical examination of patients revealed a moderate severity of symptoms (Scale for Assessment and Rating of Ataxia (SARA) [6]) and significant gait impairments (Functional Gait Assessment (FGA) [7] (Table 1).

**Table 1:** Clinical characteristics and ambulatory functions of the patients

Demographic characteristics	
N	18 (9 women)
mean age (years)	58.83 ± 12.0
height (m)	1.73 ± 0.07
weight (kg)	72.6 ± 6.1
duration of symptoms (months)	72.0 ± 29.3
median SARA score [range]	12 [8; 20]
Etiology of ataxia	
sporadic	10x SAOA, 2x MSA-C
hereditary	2x ADA, 1 x CACNA-1A mutation, 2x SCA 2, 1x SCA 1
Walking performance	
median FGA score [range]	19 [6; 30]
Ambulatory status	
independent	12
Intermediate use of aides	4
prevalent use of aides	2

Legend: *Abbreviations*: *FGA* Functional Gait Assessment [7], *SARA* Scale for the Assessment and Rating of Ataxia [6], *SAOA* Sporadic Adult Onset Cerebellar Atrophy, *ADA* Autosomal Dominant Ataxia, *CACNA*-*1A* Calcium Channel. voltage-dependent Alpha 1A subunit (P/Q type), *SCA* Spino Cerebellar Ataxia MSA-C multiple system atrophy with cerebellar features

**Table 2:** Patient characteristics, categorized by patient number, gender, age, etiology, duration of disease, neuro-ophthalmological findings, participation in a previous case study [3] as well a clinical outcome scores before and under treatment with Acetyl-DL-leucine

*ID*	*Age*	*ataxia type*	*duration*	*neuro-ophthalmological findings*	*SARA pre*	*SARA post*	*Study*
	*gender*						*Strupp et al. 2013* [3]
1	73, m	SAOA	>24	8	12.5	9.5	
2	57, w	ADA	>120	1, 3	8.0	6.0	X
3	73, w	SAOA	24	1, 2, 4	20.0	15.0	
4	54, w	MSA-C	36	1, 2, 5, 8	13.0	12.0	
5	59, m	SCA 2	56	1, 2, 7, slow saccades	16.0	12.5	
6	23, m	SCA 2	36	1, 2, 7, slow saccades	13.0	9.0	X
7	68, w	ADA	>120	1, 2, 3, 6, 7, 8, hypometric saccades	17.0	11,0	X
8	60, m	SAOA	24	1, 6	14.0	12.5	X
9	68, w	MSA-C	36	1, 2	10,0	9.5	
10	54, m	SAOA	60	1, 2, 6, 8	12.0	9.5	
11	63, w	SAOA	>120	1, 2, 3, 4, 6, 7	14.5	12.5	X
12	51, m	SAOA	36	1, 2, 3, 6, 7 hypometric saccades	14.0	12.0	X
13	51, w	EA 2	24	1	17.0	16.0	
14	49, m	SAOA	36	1, 2, 6, 7	16.5	15.5	
15	63, w	SAOA	>120	1, 2, 3, 4, 6, 7	11.5	7.0	X
16	67, m	SAOA	48	1, 2, 4	13.5	11.5	
17	60, m	SAOA	36	1, 3, 5, 8	16.0	12.5	
18	47, w	SCA 1	36	1, 2, 5 (bilateral), 6, 7, hypermetric saccades	17.0	9.5	X

Legend: *Abbrevations*: m man, w woman, SCA spino cerebellar ataxia, EA 2 episodic ataxia type 2, MSA-C multiple system atrophy with cerebellar features, *SAOA* sporadic adult-onset ataxia of unknown etiology, *ADA* autosomal dominant cerebellar ataxia, 1 saccadic smooth pursuit, 2 gaze-evoked nystagmus, 3 head-shaking nystagmus, 4 rebound nystagmus, 5 pathological head-thrust test (uni- or bilateral), 6 impaired visual fixation suppression of the VOR, 7 pathological optokinetic reflex, 8 downbeat nystagmus

**Fig. 1:**
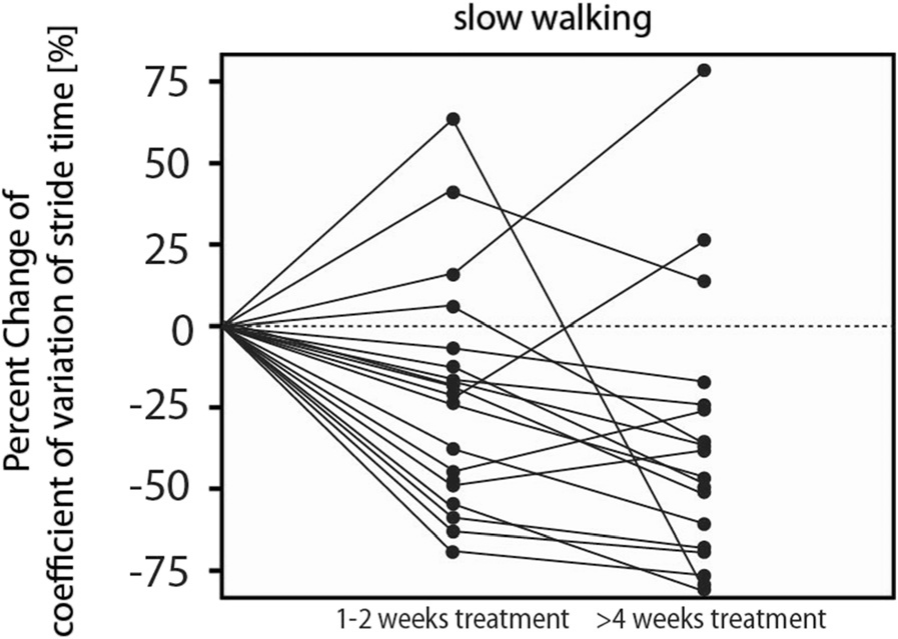
Percent change of the coefficient of variation (CV) of stride time under continuous treatment with Acetyl-DL-leucine. Legend: Scatter plot with individual courses of temporal gait variability before and under treatment with Acetyl-DL-leucine.14 patients showed a reduction of CV of stride time by 36 % during 1-2 weeks of treatment (1-2 weeks) and 49 % during > 4 weeks of treatment

Compared to the baseline measurement, walking speed was not significantly different under treatment at 1-2 weeks or at >4 weeks of treatment. For CV of stride time, we found a significant reduction in the rmANOVA model (df = 2, *F* = 6.64, *p* < .01), in the posthoc analysis only for the condition of slow walking (*F* = .3.41, *p* < .05). Here, 14 patients showed a reduction of CV of stride time by 36 % during 1-2 weeks of treatment (1-2 weeks) and 49 % during > 4 weeks of treatment (Fig. 1).

Subjective ambulatory scores (FES-I and ABC) were also improved in 12 patients, all of which showed a reduction of temporal gait variability. The SARA score showed an improvement of the median from 13 to 11 points (*F* = 3.25, *p* < .05) (for further information see Table 2). This effect did not correlate with the change of the CV of stride time.

## Discussion

In summary, Acetyl-DL-leucine reduced temporal gait variability in 14 out of 18 patients with sporadic or hereditary CA. Acetyl-DL-leucine may thus offer a new and complementary symptomatic treatment option for cerebellar gait disorders.

The improvement of variability was restricted to the condition of slow walking, where walking stability is thought to critically rely on the sensory integration function of the cerebellum [8, 9]. In this context it is important to note that instabilities at slow walking are highly associated with a history of falls in patients with CA [2]. In animal models, Acetyl-DL-leucine has been shown to interact with structures of central vestibular function [10, 11]. An FDG-PET-study in humans demonstrated increased glucose metabolism in responders to Acetyl-DL-leucine treatment in central sensorimotor integration centers [12]. Thereby it may indirectly influence sensory processing of the cerebellum for maintaining dynamic stability during walking. We did not find a correlation of gait variability improvement with the improvement of clinical scores (FGA, SARA), although the SARA score did show a significant improvement under treatment. Taking together the present findings and a recent study on the improvement of gait variability under treatment with 4-aminopyridine [13, 14], support the idea that specific functions of walking might be influenced by pharmacotherapy. These findings promote future randomized placebo-controlled, double-blinded clinical trials on the symptomatic treatment of CA, such as the FACEG (Fampridine) and the ALCAT trial (Acetyl-DL-leucine).

Analysis of speed-dependent effects on gait variability showed that non-preferred slow (acetyl-DL-leucine) and fast (4-aminopyridine) walking speeds are most responsive to pharmacological treatment in patients with CA. Whereas aminopyridines have been shown to improve gait variability mainly during fast walking [13, 14], Acetyl-DL-leucine was effective in reducing gait variability during slow walking. These findings are in accordance to an earlier study of our group showing the amount of gait variability being highest during slow and fast walking modes [8]. Thus, future randomized, controlled trials should take the speed-dependency of gait variability in patients with CA in account. Moreover, it appears to be questionable whether the clinical assessment of gait performance as determined in the SARA is sufficient and sensitive enough to assess improvements of dynamic stability in patients with CA during therapeutic interventions, since slow walking modes are commonly not examined.

If considering the assessment of dynamic stability during walking, study protocols should also include prospective recording of falls and the examination of gait variability markers.

### Consent

Written informed conset was obtained from the patients for publication of the manuscript, accompanying images and figures.
